# Simple headache revealed a rare lymphoma: Waldenstrom macroglobulinemia with unique markers: a case report and review of the literature

**DOI:** 10.1186/s43046-022-00107-6

**Published:** 2022-03-07

**Authors:** Ahmed K. Awad, Merihan A. Elbadawy, Maty Boury, Amanda Rivera, Karam Motawea, Jaffer Shah, Shanli Parnia, Joseph Varney

**Affiliations:** 1 Faculty of Medicine, Ain-Shams University, Cairo, Egypt; 2 American University of the Caribbean, School of Medicine, Philipsburg, St. Maarten SXM; 3 Faculty of Medicine, Alexandria University, Alexandria, Egypt; 4 Drexel University College of Medicine, Philadelphia, USA; 5 Morehouse School of Medicine, Atlanta, Georgia United States

**Keywords:** Waldenstrom macroglobulinemia, Headache, Lymphoma, Monoclonal immunoglobulins

## Abstract

**Background:**

Waldenstrom macroglobulinemia (WM) is a rare lymphoma with an incidence rate of 3 per million people per year, with approximately 1000 to 1500 new cases diagnosed each year in the USA. It is primarily seen in Caucasian males with a median age of 70 years old. Patients are most often asymptomatic, but WM can manifest itself with constitutional symptoms such as lethargy, bleeding, organomegaly, and neurological or fundoscopic abnormalities. WM is characterized by immunoglobulin M (IgM) monoclonal gammopathy, lymphocytic infiltration of bone marrow, and normocytic anemia due to bone marrow replacement.

**Case presentation:**

Our patient is a Hispanic 67-year-old female that presents with one month of intermittent band-like bilateral headache accompanied by dizziness, light-headedness, nausea, and blurred vision. A thorough diagnostic workup was performed, including serum protein electrophoresis (SPEP) with serum immunofixation (SIFE) showing an M spike and IgM kappa. Bone marrow biopsy was significant for lymphoplasmacytic infiltration with nodular B cells (CD19+, CD20+, CD22+). Computerized Tomography (CT) imaging showed splenomegaly in the patient. Treatment was provided for hyperviscosity syndrome with plasmapheresis twice. The patient reported improvement of her symptoms and was then scheduled for chemotherapy. Throughout 7 months, our patient received multiple cycles of bortezomib, dexamethasone, and rituximab. While her symptoms improved her psychiatric status got progressively worse.

**Conclusion:**

It is important not to neglect symptoms such as a headache, which may seem small, but could serve as a clue in the diagnosis of Waldenstrom's macroglobulinemia.

## Background

In 2003, the World Health Organization described Waldenstrom macroglobulinemia (WM) as a lymphoplasmacytic lymphoma associated with a monoclonal immunoglobulin M (IgM) protein [1]. In 1958, Waldenstrom initially diagnosed WM in two patients presenting with nasal bleeding, lymphadenopathy, anemia, thrombocytopenia, and elevated ESR [2]. WM is a lymphoplasmacytic lymphoma with a monoclonal pentameric IgM protein. Expressing pan B cell markers (e.g., CD19 and CD20) and typically test negative for CD3 and CD103, pleomorphic B-lineage cells at different maturation stages invade the lymph nodes and bone marrow [3].

Being diagnosed by the presence of 10% clonal lymphoplasmacytic cells in BM, WM is usually complicated with Multiple myeloma and rarely complicated with renal failure or nephrotic syndrome, yet renal failure can present with microhematuria or mild proteinuria. In our case we describe a case of WM presenting with only headaches and blurred vision.

## Case presentation

In June 2016, a 67-year-old hypertensive female with chronic hyperlipidemia, controlled by a statin, presented with a headache and blurred vision. O/E she was afebrile, 64 bpm, 161/91 mmHg. The patient states that the headache was intermittent for the past month but persisted for three days. It is band-like in quality, bilateral and exacerbated by light and movement, and is associated with dizziness, light-headedness, and nausea. The patient denied vomiting or diarrhea. She has seen an ophthalmologist and had planned to undergo a cataract extraction procedure with intraocular lens insertion. Worsening and blurred vision in conjunction with the headache was also reported. She was on Tylenol 1000 mg IV, Toradol 30 mg, and Prednisone 60 mg once per day. The patient’s past surgical history includes two C-sections, and her past familial history consisted of a sister diagnosed with breast and pancreatic cancer.

**Fig. 1:**
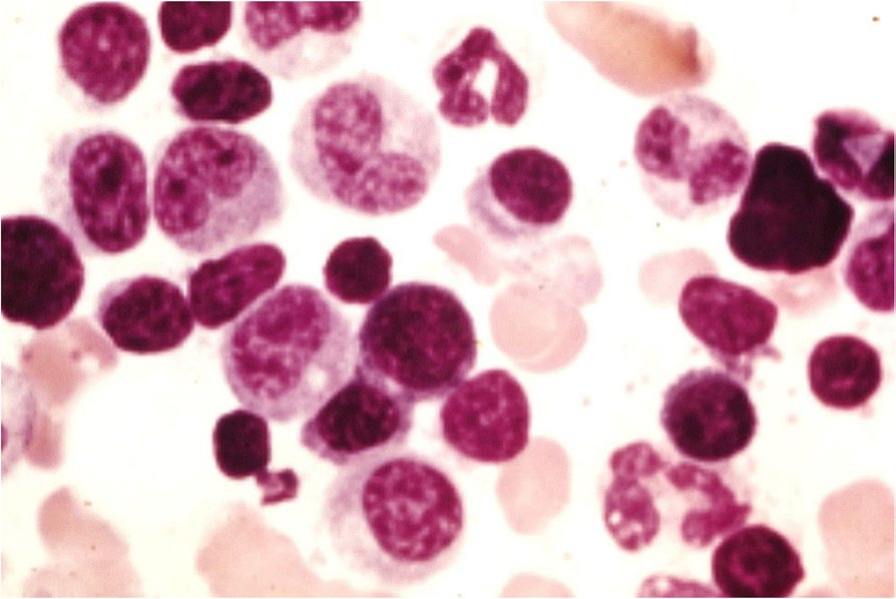
Microscopic imaging

**Fig. 2:**
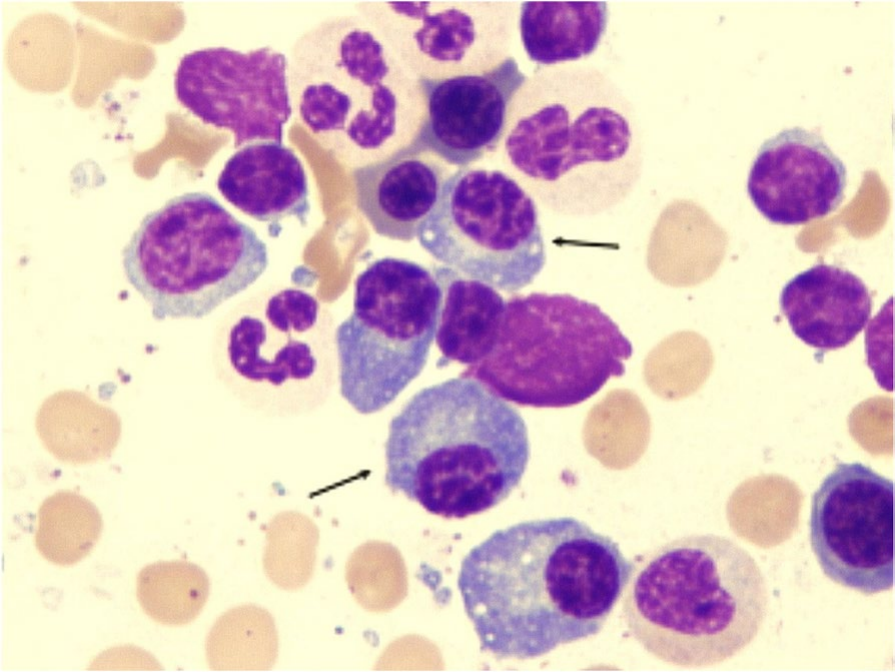
Microscopic imaging

**Fig. 3:**
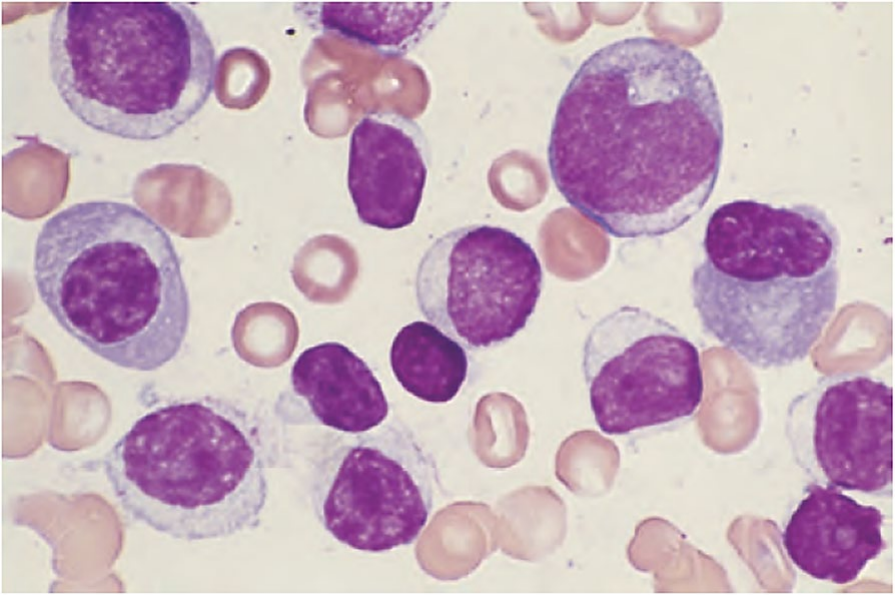
Microscopic imaging

Laboratory tests yielded elevated serum protein, after which serum protein electrophoresis with Immunofixation electrophoresis (IFE) of blood was performed. This showed an M spike and Ig M Kappa. The patient was treated for clinical hyperviscosity syndrome and reported improvement in her symptoms. Plasmaphereses resulted in a significant decrease of IgM from 3900 mg to 900 mg. The patient was scheduled for BM biopsy, which showed micronodular infiltration of lymphoid cells with phenotype CD20 CD5-(90%), CD 138 (10%), Furthermore, histopathology declared nodular B cell and lymphoplasmacytic infiltrate, positive for CD 20, CD 79a, and PAX -5, yet negative for CD 10. Cytogenetics showed 46 XX, FISH positive for deletion 1p, negative for immunoglobulins abnormalities, and p 53. Thus, the findings were consistent with the international criteria for the diagnosis of lymphoplasmacytic lymphoma (WM) (Figs. 1, 2, and 3).

Radiographic imaging of the bones and a total body computed tomography showed splenomegaly alone yet did not show any other lytic lesions, organomegaly, or lymphadenopathy. PET-CT scan was negative for any abnormal metabolic activity. Over seven months, our patient received multiple cycles of Bortezomib, Dexamethasone, and Rituximab. Her M-spike was no longer detectable (2.7 g/dL on admission), her IgM decreased to 76 mg/dL (3900 mg/dL on admission). Her repeat abdominal CT showed resolution of splenomegaly, and she no longer experienced headaches or visual disturbances. Our patient’s treatment was extremely taxing on her emotional well-being resulting in depression, anxiety, hopelessness, and she was then started on psychotherapy. This calls into how we can lessen the burden of Waldenstrom macroglobulinemia treatment while still maintaining a high rate of successful outcomes, as presented in this case report.

## Discussion

Though WM is commonly asymptomatic, clinical manifestations are well reported in the literature. The most common presentation is fatigue-related to normocytic anemia, in which the median Hb value is 10 g/dL [4], with other common symptoms including hepatomegaly (20%), splenomegaly (15%), and lymphadenopathy (15%). The reason behind these clinical presentations lies behind the circulation, tissue deposition, and autoimmune properties of IgM monoclonal proteins leading to hyperviscosity. Manifested hyperviscosity has been shown in roughly 15% of cases and with multiple neurological abnormalities, visual defects, and epistaxis [5]. WM patients have not reported before in literature with only headache or blurred as in our case.

Other less common presenting symptoms include cryoglobulinemia and cold agglutinin hemolytic anemia (5%), peripheral neuropathies (5–10%), and amyloidosis (2%). Extramedullary involvement sites have also been sighted through histological examination. Pei Lin et al. [6] stated that lymphoplasmacytic lymphoma was the most common histological type, in 40 (78%) samples from total 51 specimens obtained from lymph nodes (*n*=36), soft tissue (*n*=4), spleen (*n*=3), skin (*n*=2), lung (*n*=2), tonsils (*n*=1), colon (*n*=1), liver (*n*=1), and gallbladder (*n*=1). With only one pancreatic involvement in literature, WM affects the gastrointestinal tract, most commonly the stomach [7]. Furthermore, associated psychiatric complications to WM are still unclear, yet it can be attributed to Bing-Neel syndrome, in which the central nervous system is infiltrated by WM malignant clones complicating WM case with psychiatric symptoms as happened in our case [8].

Although clonal monoclonal gammopathy of undetermined significance MGUS B cells already contain the malignant molecular signature, all patients have an earlier phase of IgM (MGUS) that passed unnoticed. With median survival for patients under the age of 70 of 10 years or more, WM has lower median survival in older age groups, approximately 7 and 4 years for those 70–79 and those 80 or older, respectively [9] and with an incidence rate higher in males than females with 0.92 per 100,000 person-years to 0.30 [10].

WM diagnosis depends on both non-invasive and invasive procedures. Non-invasive measures mainly consist of serum and 24-h urine collection for protein electrophoresis, Serum immunofixation to confirm the IgM heavy and light chains, and quantitative test for immunoglobulin G immunoglobulin A and IgM. Invasive measures: Bone marrow biopsy which displays intertrabecular monoclonal lymphoplasmacytic infiltrate ranges from predominantly lymphocytic cells to overt plasma cells. As other associated symptoms should not be denied, computed tomography (CT) of the abdomen and pelvis must be done to detect organomegaly, lymphadenopathy, and serum viscosity is required when signs and symptoms of hyperviscosity syndrome are manifested or when IgM > 4000 mg/dL.

Low volume lymphoma or hematological malignancies medical center along with inaccuracy estimate of WM incidence and prevalence are the main reasons behind the many undiagnosed WM cases despite the vast amount of cancer registry data provided. Moreover, the reason lies behind the rarity of the disease, in addition to other significant limitations. The absence of the differentiation among IgM monoclonal gammopathy of asymptomatic WM and symptomatic WM requiring therapy treatment is the most critical limitation [11]. Diagnostic criteria and method modifications that occurred over the past 30 years are also part of the limitations and inaccuracy of the retrospective registries [11]. With the continuous change in the diagnostic criteria, the treatment guidelines are in constant evolution. Yet, the main lines of treatment contain two main core treatments: treating the associated symptoms, especially hyperviscosity syndrome, and reducing tumor mass.

Although affecting a decreasing portion of patients nowadays, hyperviscosity syndrome is rarely symptomatic, it makes treatment challenging. Symptoms such as epistaxis, gingival bleeding, visual changes due to retinal hemorrhage, and central nervous system findings, including dizziness, light-headedness, and generalized fatigue, must be suspicious, and serum viscosity must be measured [12]. When hyperviscosity exists, plasma exchange should be carried out as a mainstay measure until systemic therapy successfully reduces the tumor mass and decreases the IgM protein serum concentration [12]. Variety of systemic chemotherapy to reduce tumor mass has been proposed in the literature and with supporting evidence. Lacking long-term toxicity and impact on the peripheral blood stem cells’ mobilization, Rituximab has shown the most significant impact in the treatment of WM. Drug utility is further increased when combined with ofatumumab, another monoclonal antibody [13].

Furthermore, many clinical trials have proposed the combinations between different interventions and even suggested using corticosteroids from their role in managing the inflammatory response. As for now, 4 different preferred standards of therapy are available for WM: (1) bendamustine and rituximab, (2) bortezomib and dexamethasone and rituximab, (3) ibrutinib ± rituximab, and (4) rituximab and cyclophosphamide and dexamethasone. Additional clinical studies comparing the response rates, tolerability, and the cost of these regimens are needed to aid physicians in tailoring the therapy to their patients according to the provided treatments such as carfilzomib, cladribine, and fludarabine are required to assist physicians in tailoring the treatment to their patients [14, 15].

## Conclusion

WM is a rare malignancy with a poor prognosis. With the advancement of screening tools, its diagnosis and treatment have become more manageable. With the advances of clinical trials, its treatment becomes feasible with little or no adverse effects. We present the only case in the literature of WM patients presented only by headache and blurred vision. We recommend physicians to have high yield dependence on BM biopsy and CT scans when WM is suspected.

## Data Availability

All data generated or analyzed during this study are included in this published article and its supplementary information files.

## Abbreviations

WM: Waldenstrom macroglobulinemia
SPEP: Serum protein electrophoresis
IFE: Immunofixation electrophoresis
